# PFKFB3 in neovascular eye disease: unraveling mechanisms and exploring therapeutic strategies

**DOI:** 10.1186/s13578-024-01205-9

**Published:** 2024-02-10

**Authors:** Peiyu Liu, Dandan Sun, Shuchang Zhang, Shimei Chen, Xiaoqian Wang, Huiming Li, Fang Wei

**Affiliations:** 1 Department of Ophthalmology, Shanghai General Hospital, Shanghai Jiao Tong University School of Medicine, Shanghai, China; 2 Shanghai Key Laboratory of Ocular Fundus Diseases, Shanghai Engineering Center for Visual Science and Photomedicine, National Clinical Research Center for Eye Diseases, Shanghai Engineering Center for Precise Diagnosis and Treatment of Eye Diseases, Shanghai, 200080 China; 3 Shanghai General Hospital, Shanghai Jiao Tong University School of Medicine, Shanghai, China

**Keywords:** PFKFB3, Ocular angiogenesis, Glycolysis, Endothelial cells, Oxidative stress

## Abstract

**Background:**

Neovascular eye disease is characterized by pathological neovascularization, with clinical manifestations such as intraocular exudation, bleeding, and scar formation, ultimately leading to blindness in millions of individuals worldwide. Pathologic ocular angiogenesis often occurs in common fundus diseases including proliferative diabetic retinopathy (PDR), age-related macular degeneration (AMD), and retinopathy of prematurity (ROP). Anti-vascular endothelial growth factor (VEGF) targets the core pathology of ocular angiogenesis.

**Main body:**

In recent years, therapies targeting metabolism to prevent angiogenesis have also rapidly developed, offering assistance to patients with a poor prognosis while receiving anti-VEGF therapy and reducing the side effects associated with long-term VEGF usage. Phosphofructo-2-kinase/fructose-2,6-bisphosphatase 3 (PFKFB3), a key enzyme in targeted metabolism, has been shown to have great potential, with antiangiogenic effects and multiple protective effects in the treatment of neovascular eye disease. In this review, we summarize the mechanisms of common types of neovascular eye diseases; discuss the protective effect and potential mechanism of targeting PFKFB3, including the related inhibitors of PFKFB3; and look forward to the future exploration directions and therapeutic prospects of PFKFB3 in neovascular eye disease.

**Conclusion:**

Neovascular eye disease, the most common and severely debilitating retinal disease, is largely incurable, necessitating the exploration of new treatment methods. PFKFB3 has been shown to possess various potential protective mechanisms in treating neovascular eye disease. With the development of several drugs targeting PFKFB3 and their gradual entry into clinical research, targeting PFKFB3-mediated glycolysis has emerged as a promising therapeutic approach for the future of neovascular eye disease.

## Introduction

Glucose metabolism is the main energy source for biological body functions and it supports essential cellular functions and metabolic processes [1]. In the human body, adenosine triphosphate (ATP) is generated primarily through oxidative phosphorylation, the main energy-producing pathway. However, despite its lower ATP efficiency, glycolysis contributes to the energy metabolism of specific glycolysis-dependent cells. Many cells in the retina such as endothelial cells (ECs) are highly glycolytic and are very sensitive to metabolic changes, and metabolic changes can also affect their function [2].

The regulation of glucose metabolism can be approached from various perspectives, such as the end of sugar supply, glucose metabolites and key enzymes of glucose metabolism, and targeting key enzymes of glycolysis is the direction explored by many researchers. Among the many key enzymes, PFKFB3 plays a very important role in regulating the activity of phosphofructokinase-1 (PFK-1), a key enzyme in the glycolytic pathway [3], which promotes glucose metabolism to pyruvate and generates energy. PFKFB3 regulates PFK-1 activity by synthesizing and degrading fructose 2,6-bisphosphate (F2,6BP). F2, 6BP is a strong activator of PFK-1, prompting PFK-1 to become more active, leading to an accelerated rate of the glycolytic pathway and increased ATP production. PFKFB3 also plays a key role in many other physiological and pathological processes, including vascular and metabolic diseases.

Ocular neovascular eye disease is closely related to angiogenesis and metabolic disorders [4] and is usually caused by chronic diseases, hyperglycemia, hypertension, hypoxia, and other factors, all commonly leading to retinal damage. Neovascularization may lead to ocular retinal bleeding, leakage, and even scar formation, which ultimately affects the patient’s vision [5]. There are many common neovascular eye diseases, such as PDR [6], AMD [7], and ROP [8]. The conventional treatment for these diseases is based on anti-vascular endothelial growth factor (VEGF) [9]. VEGF, which stands for vascular endothelial growth factor, is a specific proangiogenic factor that plays a pivotal role in various neovascular eye diseases, ultimately leading to blindness on a global scale [10]. VEGF overexpression can promote pathological angiogenesis by enhancing the migration and proliferation of ECs and increasing vascular permeability. This process, in turn, is a crucial target for inhibiting retinal neovascularization (RNV).

Currently, anti-VEGF treatment is widely recognized as an effective therapeutic strategy for managing neovascular ophthalmopathy in clinical practice. By administering anti-VEGF drugs through vitreous injections, vascular leakage and angiogenesis can be attenuated, ultimately improving the visual acuity of the affected patients [11]. However, recent years, there have been many controversies about anti-VEGF therapy, such as the need for frequent and long-term treatment, poor results, drug resistance, high economic burden for the patients, and many other defects [12–14]. Therefore, the development of new therapies is urgently needed.

As an emerging therapy, targeting PFKFB3 can play a significant role in neovascular eye disease. ECs and glial cells are involved in the main pathophysiological processes in neovascular eye disease, and these processes are glycolysis dependent. Targeting PFKFB3 manipulates PFKFB3-mediated glycolysis and significantly influences the function and mechanism of highly glycolytic cells. Inhibition of PFKFB3 can inhibit pathological neovascularization and glial cell activation and can also produce fewer side effects than inhibition of VEGF [15]. Our review focuses on retinal glucose metabolism patterns, physiopathological ocular angiogenesis processes, the role and potential mechanisms of PFKFB3 in neovascular eye diseases, and the leading therapeutic drugs presently targeting PFKFB3. The advent of PFKFB3-targeted therapy has led to new, diverse treatments for neovascular eye disease, thereby providing prospective avenues for patient care.

## Metabolic pattern of retina

In the majority of mammalian cells, energy is supplied primarily by the large amount of ATP generated by glycolysis, the tricarboxylic acid (TCA) cycle, and oxidative phosphorylation (OXPHOS). Nonetheless, certain cells, including various cancer types, undergo a metabolic shift characterized by increased glucose uptake and subsequent production of lactic acid, which constitutes a less efficient ATP production pathway [16, 17]. This metabolic reprogramming, which occurs in cells with a normal mitochondria and oxygen supply, is called aerobic glycolysis or the Warburg effect and can be observed in many retinal cells and tumor cells [18].

Glycolysis is essential for the development of the retina and for maintaining normal physiological function. Retinal cells directly convert approximately 90% of glucose into lactate [19]. There is a glycolytic gradient between the outer and inner retinal layers with the inner retina using OXPHOS more, except for macroglia. The photoreceptor cell layer generates energy mainly by glycolysis [20], and the resulting lactate is transferred to the retinal pigment epithelium (RPE) by monocarboxylate transporter 1 (MCT1) [21]; additionally, energy is generated by aerobic oxidation, and some lactate is transferred to Müller cells. The production of lactate during this process can promote angiogenesis via the lactate/NF-κB/IL-8 pathway [22].

Moreover, ECs, retinal glial cells, nerve cells, and pericytes constitute the neurovascular unit of the eye in the retina, and retinal ECs play a crucial role in this unit. ECs rely mainly on glycolysis rather than oxidative phosphorylation to produce energy, which can reduce the effect of oxidative stress on ECs [23]. Müller cells also acquire energy mainly through glycolysis, and the resulting lactate is subsequently transported to neural cells to provide sufficient ATP to the neural cells through the tricarboxylic acid cycle [24].

Furthermore, there are multiple critical regulators of retinal glycolysis, such as hexokinase 2 (HK2), pyruvate kinase muscle isozyme M2 (PKM2), and glucose transporter 1 (GLUT1), but the key factor of interest is PFKFB3. PFKFB3 is a member of the PFKFB family and it has the highest kinase activity among the isozymes and promotes the synthesis of F2,6BP, which is one of the most vital allosteric activators of PFK-1 [25]. PFK-1 is a key enzyme in glycolysis. PFKFB3 not only controls the flux of glycolysis to affect the function and status of cells but also regulates a variety of cellular activities beyond glycolysis. PFKFB3 possesses effects that many other key glycolytic enzymes are unable to produce, and we will elaborate on the specific mechanism of PFKFB3 and its importance later.

### Physiological and pathological retinal angiogenesis mechanisms

#### Physiological retinal angiogenesis

Retinal angiogenesis is closely related to glucose metabolism, primarily due to the reliance of ECs, the key cells involved in angiogenesis, on ATP generated through glycolysis. Vascularization of the retina initially occurs in the innermost layer of the optic disc, from which it expands radially. This vascular network extends to the retinal margin before birth [26] and ultimately forms an avascular region known as the fovea.

**Fig. 1:**
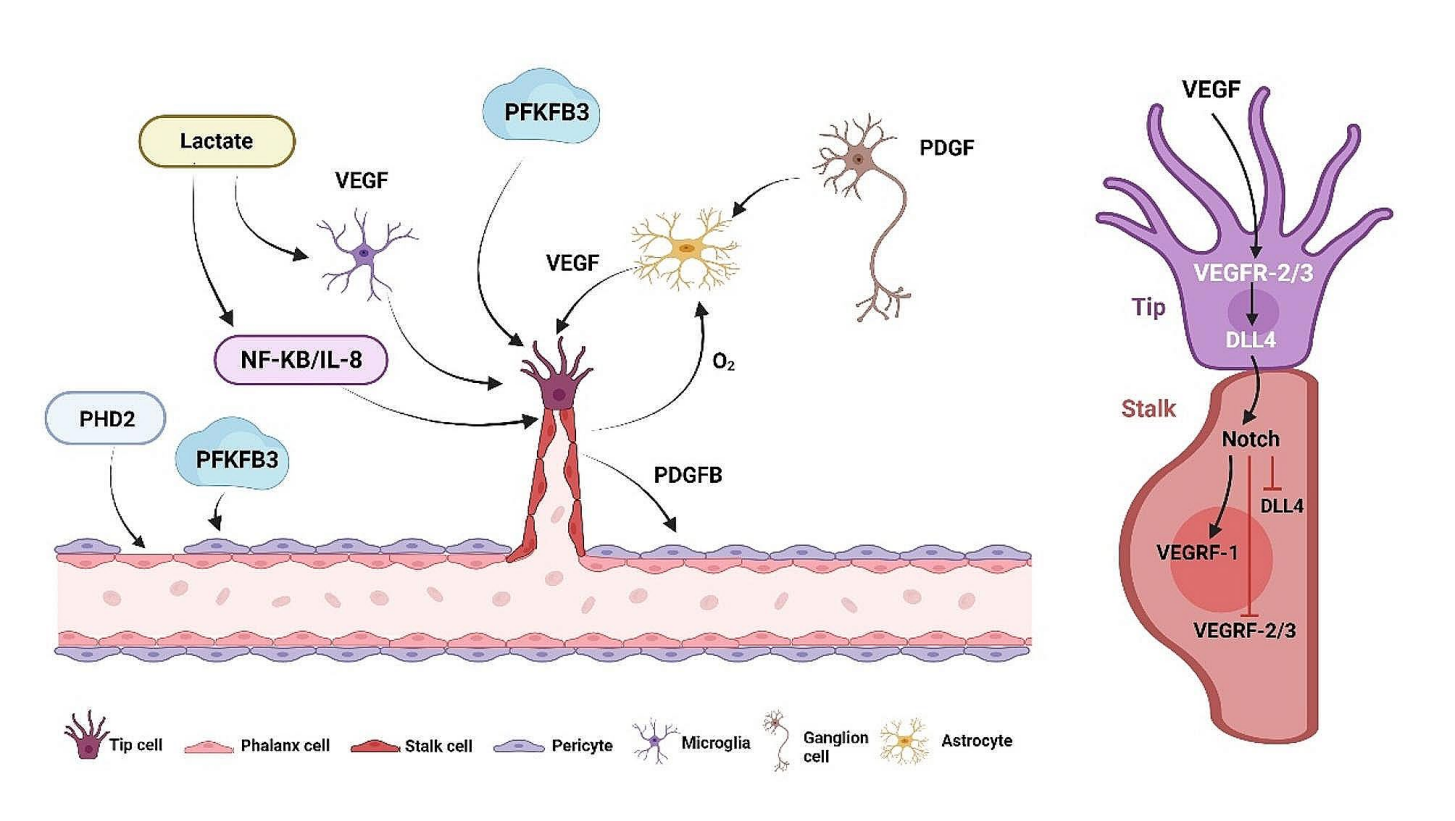
The mechanism of retinal vascular sprouting There is a complex interaction between retinal nerve cells, glial cells, and pericytes in vascular sprouting. These elements play important roles in the regulation of this process. ECs play a crucial role in the vascular system by responding to various regulators of blood vessel function and other enzymes involved in glucose metabolism. One of the most important of these proteins is the PFKFB3 protein, as well as VEGF. In the course of these reactions, ECs differentiate into three types of cells: tip cells, stalk cells, and phalanx cells. The VEGF-VEGFR and DLL4-Notch pathways regulate the transition and growth of tip cells and stalk cells. Collectively, these cells control blood vessel migration, proliferation, and integrity. The figure was created using Biorender (https://www.biorender.com/)

Angiogenic sprouts are the primary mechanism of retinal vascularization, and this process involves a variety of cells, including ECs, glial cells, and pericytes. The specific mechanism is shown in Fig. 1. ECs constitute a significant part of retinal angiogenesis, but in addition, the involvement of glial cells with pericytes is indispensable. Astrocyte precursors enter the retina from the optic nerve and grow radially to the periphery in response to nerve-derived platelet-derived growth factor (PDGF) stimulation. Blood vessels also grow along the course of astrocytes. With oxygen stimulation, astrocytes gradually migrate and mature, after which their secreted VEGF decreases [27]. This results in the establishment of a comprehensive negative feedback mechanism that curbs vascular growth.

Microglia are immunologically competent macrophages that arise along retinal vessels during retinal development and are activated in ischemic diseases. Like ECs, microglia are characterized by high glycolysis, and the findings of Liu et al. [28] suggest that the glycolytic microglia (PRAGMs) associated with pathological retinal angiogenesis not only express M1 and M2 markers but also produce a range of cytokines with proinflammatory and proangiogenic properties. Moreover, when lactate is released from ECs, it helps stimulate bone marrow-derived macrophages and retinal microglial activation and polarization [29], and interacts with retinal ECs with excessive glycolysis, resulting in pathological sprouting of ECs in proliferation.

Pericytes are smooth muscle cells found on the lateral side of vessel walls and they are closely related to the growth and formation of blood vessels. Pericytes are recruited by ECs in a platelet-derived growth factor subunit B (PDGFB)-dependent manner, leading them to attach to ECs [30]. A decrease in PDGFB activity leads to decreased pericyte coverage, causing a loss of normally growing blood vessels, abnormal angiogenesis [31], and ultimately, vascular retinopathy, such as diabetic retinopathy. Some pericytes can also promote the growth of blood vessels by secreting VEGF and they can also promote the growth of blood vessels through transforming growth factor-β1 (TGF-1) and vascular endothelial growth factor receptor-1 (VEGFR-1), which are ECs receptors [32, 33].

The most crucial cells involved in retinal angiogenesis, which promote both angiogenesis and repair, are organized as retinal ECs. ECs are divided into three primary morphologies: one tip cell always walks anteriorly, one stalk cell immediately follows, and finally a phalanx cell remains at rest [34]. The three kinds of cells are highly coordinated and cooperative during angiogenesis. At the highest VEGF concentration, some ECs are selected as tip cells to guide new vascular sprouts toward the source of growth factors by migrating [35]. During tip cell migration, blood vessels continue to lengthen in the direction of new sprouts, and a large number of stalk cells are required to complete this process. When angiogenesis is complete, the vessel walls are stable, blood flow is patent, and ECs switch to their quiescent state, phalanx cells [34], while significantly reducing the demand for and rate of glycolysis.

Despite originating from the same vessel, tip cells and stalk cells are functionally and morphologically distinct. A tip cell has many filopodia that match its high motility, whereas a stalk cell has relatively few filopodia protrusions [36]. Furthermore, the ratio of tip cells to stalk cells is dynamic. During this process, ECs compete for the tip cell position, and the EC that is most suitable for serving as a tip cell is continuously selected.

Tip cells maintain nearby ECs in the stalk cell phenotype by expressing the Notch ligand Delta-like 4 (Dll4). In adjacent ECs, Dll4 binds to the Notch receptor, resulting in the release of the Notch intracellular domain (NICD), thereby promoting increased expression of the high-affinity inducible receptor VEGFR1 while reducing vascular endothelial growth factor receptor-2 (VEGFR2) expression [37]. An increased VEGFR1/VEGFR2 ratio decreases the EC response to VEGF, leading to a stalk cell phenotype. VEGFR1 can act as a VEGF antagonist due to its decoy receptor activity, giving ECs with the lowest VEGFR1/VEGFR2 ratio (the most appropriate ECs for VEGF sensing) a tip position at the most anterior end of the bud [38]. The VEGF-Dll4-Notch signaling cascade serves as the principal regulatory mechanism for tip-stalk differentiation. DLL4 plays a pivotal role in the tip phenotype, functioning as a switch that regulates tip/stalk status. Nevertheless, this intricate process of ductal sprouting and patterning is determined collectively by other signaling pathways. Eventually, the phalanx cells connect the new blood vessels with the existing blood vessels to form a closed chamber for blood flow [39]. In this way, retinal physiological angiogenesis is achieved through the concerted efforts of various cell types, such as ECs, glial cells, and pericytes. Normally, each cell type executes its specific role, and disruption of this equilibrium can precipitate the retina into a pathological angiogenesis phase.

#### Pathological retinal angiogenesis

Pathological retinal vessels are formed in response to various factors. Pathological neovascularization is characterized by disruption of the vascular barrier, vascular leakage, and vascular dysfunction [40], leading to retinal hemorrhage and retinal detachment. There are a variety of retinal diseases characterized by pathological angiogenesis, such as diabetic retinopathy (DR), AMD, and ROP.

DR is a common complication of diabetes and is the leading cause of blindness in middle-aged and older adults, with a very high incidence in diabetic patients [41]. Therefore, we used DR as an example to elaborate on the mechanism of retinal pathological neovascularization. Vascular injury and neovascularization induced by hyperglycemia in diabetes involve several classical metabolic pathways, such as the hexosamine pathway, the polyol pathway, the formation of advanced glycation end products (AGEs), and the activation of protein kinase C (PKC) [42]. The pathophysiological changes observed in DR include various changes caused by hyperglycemia, including increased retinal vascular permeability, tissue ischemia, and neovascularization [41].

DR can be divided into two stages: nonproliferative diabetic retinopathy (NPDR) and PDR. The NPDR stage is characterized by apoptosis, gliosis, and other retinal neurodegeneration [43], and neovascularization occurs mainly in the PDR stage [44]. In PDR, due to EC dysfunction caused by hyperglycemia, neovascularization results in a lack of pericyte coverage and increased vascular leakage [44], thus further worsening the blood supply to the retina and leading to capillary occlusion as well as retinal ischemia. Hypoxia results from retinal ischemia, which triggers the production of a substantial quantity of hypoxia-inducible factor (HIF-1α). This, in turn, upregulates VEGF, leading to considerable pathological retinal angiogenesis. Consequently, patients may experience impaired vision due to frequent episodes of vitreous hemorrhage and retinal detachment.

In addition to VEGF, a variety of proangiogenic factors, such as angiopoietin (Ang) (Ang1, Ang2) [45] and fibroblast growth factor (FGF) (FGF2, FGF4) [46], are involved in the regulation of vascular permeability and angiogenesis. Ang-2 is an antagonist of endothelial receptor tyrosine kinase and can participate in the regulation of retinal vascular permeability and leakage [45]. The variety of angiogenic factors other than VEGF that are involved in the functional regulation of retinal vessels provides new insights into the treatment of retinal vascular disease.

### The role of oxidative stress in retinal diseases

According to the unified mechanism theory, long-term exposure of ECs to high glucose leads to metabolic disorders, which cause mitochondria to produce more reactive oxygen species (ROS) [47]. ROS increase deoxyribonucleic acid (DNA) damage in ECs, and this leads to excessive activation of the DNA repair enzyme poly (ADP-ribose) polymerase (PARP) [48], resulting in adverse effects, including substantial nicotinamide adenine dinucleotide (NAD) consumption and modification of glyceraldehyde-3-phosphate dehydrogenase (GAPDH). Eventually, upstream glucose metabolites enter the glycolytic pathway, as described previously, through four pathways, ultimately leading to mitochondrial damage, retinal cell apoptosis, and chronic inflammation. In addition, mitochondrial injury is caused by the upregulation of gelatin matrix metalloproteinases (MMPs) via the nicotinamide adenine dinucleotide phosphate (NADPH) oxidase (NOX) complex [49], and the dual stimulation of high glucose and oxidative stress promotes the entry of MMP2 and MMP9 into mitochondria and subsequent damage. In the NPDR stage, oxidative stress is aggravated by continuous exposure of ECs and pericytes to high glucose concentrations, resulting in increased activity of caspase-3, nuclear factor κB (NF-κB), and other factors and accelerated retinal cell death. The primary mechanism is that ROS promote mitochondrial permeability, cytochrome c (Cyct-c), and the proapoptotic factor exocrine and activate caspase-9 and caspase-3 to activate apoptotic pathways [50]. Conversely, inflammation is activated mainly by NF-κB factors, which can play proinflammatory and proapoptotic roles [51]. In addition, the expression of chemokines such as monocyte chemoattractant protein-1 (MCP-1), macrophage inflammatory protein-1 (MIP-1)α and MIP-1β, tumor necrosis factor-α (TNF-α), interleukin-6 (IL-6), interleukin-8 (IL-8) and interleukin-β (IL-1β) is significantly upregulated in diabetic patients [52], and the severity of DR is closely related to the expression of inflammatory factors.

Glial cells are concurrently implicated in the development of inflammation under conditions of high glucose stimulation. Microglia are first activated by high glucose stimulation to release proinflammatory factors [53]. Astrocytes and Muller cells further amplify the inflammatory effects of these substances.

### Effect of PFKFB3 on neovascular eye disease and ECs

**Fig. 2:**
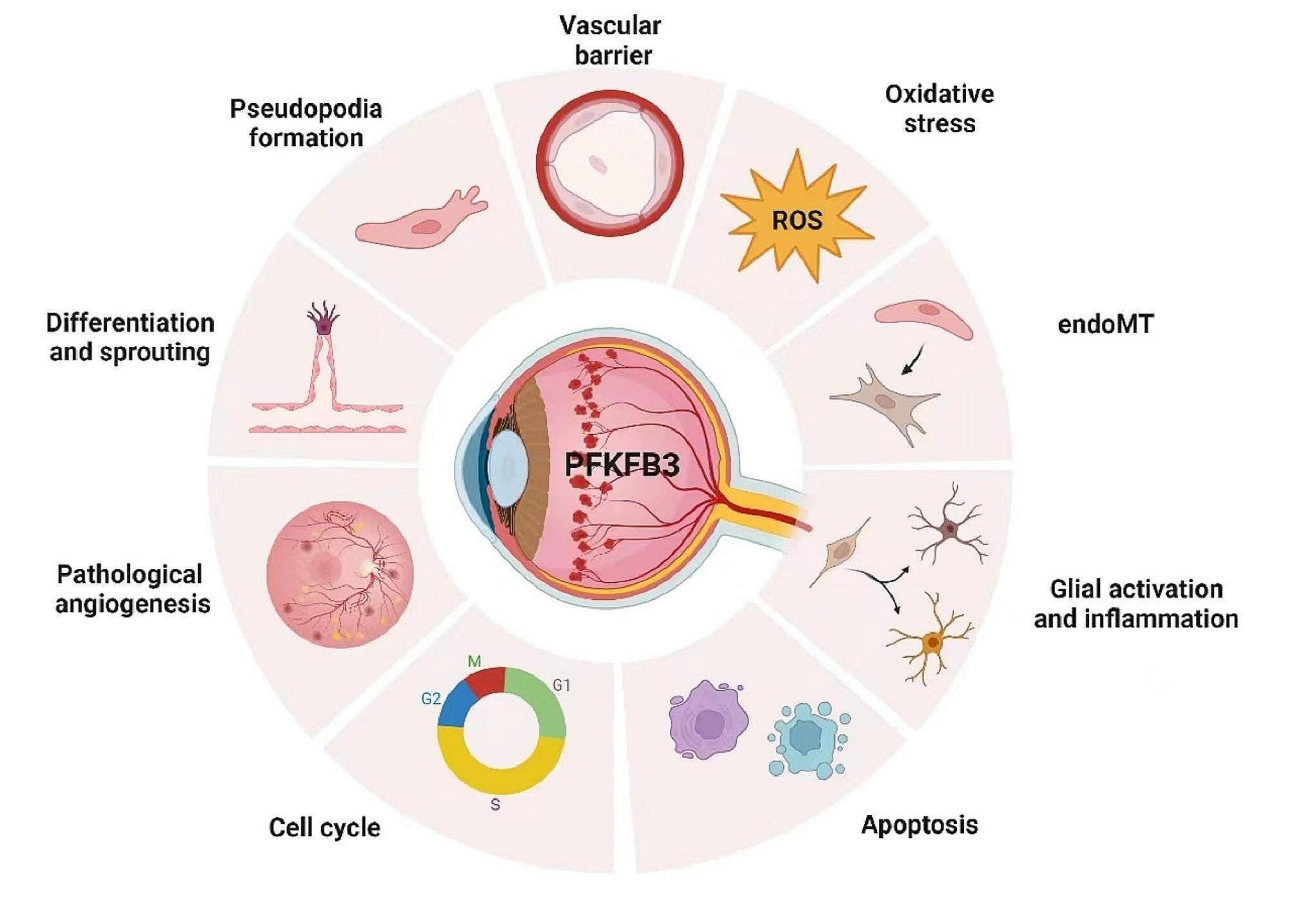
Regulatory mechanisms involved in PFKFB3 There are various regulatory mechanisms through which PFKFB3 is involved in neovascular eye diseases, and it plays a vital role in this process. This protein is involved in the differentiation of ECs, the formation of pseudopodia during physiological angiogenesis, the promotion of vascular sprouting, and the regulation of vascular barrier function during physiological angiogenesis. By reducing PFKFB3, pathological neovascularization in the eye can be inhibited. This could be achieved by targeting PFKFB3. In addition, PFKB3 has the ability to control the activation of glial cells as well as the secretion of inflammatory factors, and it contributes to various mechanisms, such as oxidative stress injury, endoMT, cell cycle regulation, and apoptosis. The figure was created using Biorender (https://www.biorender.com/)

PFKFB3 is a key enzyme in glycolysis that not only participates in EC growth and differentiation but also plays an essential role in various regulatory mechanisms in angiogenesis. As shown in Fig. 2. Moreover, PFKFB3 has been shown to act as a therapeutic target and participate in a variety of regulatory mechanisms in various diseases, such as cancer [54–57], pulmonary hypertension [58, 59], pulmonary fibrosis [60–62], and atherosclerosis [63, 64]. Moreover, neovascular eye disease has many mechanisms of action and protection.

### Effect of PFKFB3 on pathological angiogenesis

At the onset of the 20th century, diverse perspectives posited that vascular sprouting could be exclusively regulated by genetic signals, but De Bock et al.’s study [65]showed that glycolysis also controlled vascular sprouting. PFKFB3 regulates glycolysis in ECs, and the results of the present study further demonstrated that PFKFB3 controls vascular sprouting by regulating glycolysis. In a separate study, the PFKFB3 inhibitor 3PO effectively suppressed glycolytic activity in mouse ECs via oxygen-induced retinopathy (OIR) and choroidal neovascularization (CNV). This inhibition consequently led to a marked reduction in vascular growth, specifically in terms of branch formation, thereby successfully mitigating pathological angiogenesis within the ocular context [66].

Furthermore, the suppression of glycolysis amplifies the effectiveness of anti-VEGF agents, revealing a novel avenue and strategy for inhibiting angiogenesis. In addition to 3PO, recently developed PFKFB3 inhibitors, namely, PA-1 and PA-2, have been shown to demonstrate antiangiogenic effects in vitro. These inhibitors effectively impede EC proliferation and migration and suppress vascular sprouting [67], demonstrating that targeting PFKFB3 is a promising therapeutic strategy for preventing vascularization.

Others have focused on the role of adenosine A2a receptor (Adora2a) -mediated glycolysis in pathological retinal angiogenesis in ECs, and they found that Adora2a gene expression was significantly increased in the retinas of OIR mice. Adora2a promoted pathological angiogenesis by promoting the activation of glycolysis, and PFKFB3 expression was also significantly increased in OIR mice [68]. Finally, PFKFB3 inhibitors were found to reverse the pathological angiogenic effect produced by Adora2a gene overexpression in vitro, and Adora2a knockout also significantly downregulated PFKFB3 expression, ultimately resulting in reduced hypoxia-induced angiogenesis. Another study examining Yes-associated protein (YAP) [69] found that inhibiting PFKFB3 by inhibiting YAP prevented pathological angiogenesis and metabolic abnormalities and reduced the degree of retinopathy, demonstrating that targeting YAP with PFKFB3 may be a potential target for the treatment of neovascular retinal diseases.

In addition to targeting ECs, the role of macrophages in ocular angiogenesis has been investigated. Liu et al. [28] found that PRAGMs associated with pathological retinal angiogenesis were formed in response to lactate stimulation in the OIR model, and PFKFB3 was explicitly knocked down in macrophages/microglia to normally promote the proliferation and differentiation of ECs and inhibit pathological angiogenesis. Moreover, these findings demonstrated that there was a close interaction between glia and ECs. A recent study also inhibited laser-induced CNV formation in mice by specifically knocking down PFKFB3 in macrophages/microglia [70], and AZ67, an inhibitor of PFKFB3, again verified this mechanism. Overall, targeting PFKFB3 has demonstrated favorable outcomes in both in vitro and in vivo experiments, suggesting that targeting PFKFB3 is a promising therapeutic strategy for neovascular eye diseases.

### Effect of PFKFB3 on cell differentiation and vascular sprouting in ECs

In the preceding discussion, we elucidated the mechanism by which the epidermal growth factor (EGF)-Dll4-Notch signaling pathway predominantly governs the differentiation of ECs in physiological states as well as during vascular sprouting [34]. Dll4 is a signaling molecule that induces the tip cell phenotype while enhancing Notch1 signaling in neighboring stalk cells. Concurrently, this treatment suppresses the expression of PFKFB3 in stalk cells. Inhibition of PFKFB3 expression diminishes tip cell competitiveness and vascular sprouting. Conversely, the overexpression of PFKFB3 counteracts the inhibitory impact of Notch signaling on vascular sprouting, fostering the tip cell phenotype and promoting vascular sprouting [65]. PFKFB3 plays a crucial role in the competition and differentiation between tip cells and stalk cells and can participate in the regulation of tip/stalk cells together with other genetic factors.

Most ECs, such as tip cells and stalk cells, have high glycolytic activity [71], and although PFKFB3 increases glycolytic flux while promoting the expression of the tip cell phenotype, it has been demonstrated that tip cells have a lower glycolytic capacity than nontip cells and a greater ability to respond to metabolic changes in human umbilical vein endothelial cell (HUVEC) cultures [72]. Although phalanx cells are quiescent, they maintain their basal glycolytic capacity [73]. Glycolysis is an essential process for the differentiation of tip cells. In a recent investigation, it was discovered that inhibition of PFKFB3 mRNA does not affect the proportion of tip cells within the EC population [74]. However, it does induce the tip cell phenotype in the nontip cell population. Furthermore, researchers have shown that silencing PFKFB3 has no discernible impact on glucose uptake in HUVECs. PFKFB3 regulates the phenotypic selection of ECs and demonstrates that metabolism continuously affects the differentiation and sprouting of ECs.

### Effect of PFKFB3 in pseudopodia formation

During EC motility, the glycolytic enzyme PFKFB3 localizes to lamellipodia, which are formed by ECs enriched with F-actin. Additionally, it is present in the cell body but is excluded from filopodia. This localization results in the generation of significant amounts of ATP within membrane folds that are enriched with F-actin [65]. F-actin interacts with PFKFB3 to enhance PFKFB3 activity, increase glycolytic flux with ATP generation, and form “ATP hotspots”. Conversely, silencing PFKFB3 impairs the formation of filopodia and lamellipodia, and recent studies have shown that manipulation of PFKFB3 inhibits the formation of filopodia and lamellipodia in ECs [75–77].

### Effect of PFKFB3 in vascular barrier

Healthy blood vessels possess a normal vascular barrier, and the quality of the vascular barrier is closely related to VE-cadherin expression [78]. This essential adhesion protein plays a role in vascular rearrangement and is indispensable for the growth and angiogenesis of ECs. PFKFB3 does not affect VE-cadherin expression but rather affects its migration and turnover rates, thereby interfering with its adhesion [76]. In a separate investigation, the upregulation of VE-cadherin was observed through the inhibition of PFKFB3 in both in vivo tumor models and in vitro HUVEC cultures. This phenomenon contributes to the fortification of the vascular barrier [79]. However, the impact of targeting PFKFB3 on the vascular barrier in human retinal microvascular ECs (HRMECs) and specific ocular disease models needs further validation and exploration.

### Effect of PFKFB3 on oxidative stress damage

Long-term hyperglycemia causes abnormal glucose metabolism in the retina and glucose influx into the glycolytic bypass [80], which leads to increased production of ROS as well as oxidative stress damage in ECs. This process is the main mechanism of DR, and targeting glucose metabolism is also a significant treatment for coping with oxidative stress. In a recent study by Sun et al., oxidative stress in diabetic retinopathy (DR) mice was alleviated by knockdown of forkhead box protein O1 (FOXO1) and overexpression of PFKFB3 [81]. These researchers alleviated oxidative stress damage in ECs caused by high glucose by overexpressing PFKFB3 through interaction with the Mre11-Rad50-Nbs1 (MRN)-ataxia telangiectasia mutated (ATM) pathway, which promotes DNA damage, vascular leakage in the retina, and cellular senescence. Retinal ganglion cells (RGCs) in the retina form the optic nerve and are important components of the visual system. RGCs are also susceptible to oxidative stress, such as ischemia and hypoxia, as well as hyperglycemia, and are also highly dependent on oxidative phosphorylation [82–84]. However, in RGCs, overexpression of PFKFB3 leads to elevated oxidative stress, causing irreversible damage to the RGCs [85]. The impact of PFKFB3 thus varies across distinct cell types and under diverse pathological conditions. Further investigations are warranted to elucidate the specific underlying mechanisms involved.

### Effect of PFKFB3 on endothelial mesenchymal transformation (endoMT)

Neovascularization and fibroproliferative membrane formation are pathological hallmarks of proliferative diabetic retinopathy (PDR), and a large amount of extracellular matrix (ECM) and abundant myofibroblasts accumulate in fibrotic tissue. In the retina of PDR patients, many myofibroblasts may arise from the transformation of HRMECs, which is called endoMT [86]. Targeting the endoMT process at the same time can reduce the damage caused by DR [87]. In a recent study, investigators suppressed endoMT and fibrotic responses by inhibiting glycolysis, which was dominated by PFKFB3 in cardiac ECs. Conversely, the overexpression of PFKFB3 not only promoted endoMT but also exacerbated fibrotic responses [88]. In this study, the authors achieved endoMT inhibition in a mouse CNV model by inhibiting YAP, and subretinal fibrosis was significantly relieved [89]. According to our previous study [69], YAP is an upstream molecule of PFKFB3, and inhibition of YAP can significantly inhibit the expression of PFKFB3. Therefore, we speculate that targeting PFKFB3 can play a role in the endoMT phenotype in neovascular eye disease, but the underlying mechanism is worth exploring.

In the context of fibrosis, PFKFB3 is not only closely related to endoMT but also indirectly involved in the epithelial–mesenchymal transition (EMT) of retinal pigment epithelial cells (RPE). A recent study suggested that TGF-β2 can induce the inhibition of PGC-1α, promoting the inhibition of mitochondria and glycolysis, as well as the upregulation of key glycolytic enzymes such as PFKFB3 [90]. This cascade of events leads to the development of EMT and retinal subfibrosis.

### Effect of PFKFB3 on glial activation and inflammation

The presence of three glial cell types (Müller cells, astrocytes, and microglia) in the retina is glycolysis dependent; thus, glial activation is also tightly linked to the flux of glycolysis. Whereas microglia are highly glycolytic and inflammatory agent secretors, Wang et al.‘s study revealed that targeted knockdown of PFKFB3 inhibited amyloid β (Aβ)-mediated microglial activation, leading to inflammation and RPE damage [91], adding a strategy for the treatment of AMD. In a separate study pertaining to AMD, the targeting of PFKFB3 exhibited therapeutic effects by mitigating CNV damage and suppressing the activation of the inflammatory factor pathway [70], again verifying that controlling PFKFB3-mediated microglial activation is an effective treatment for AMD. PFKFB3 is also involved in modulating the interaction between microglia and ECs, and microglia switch to a proangiogenic phenotype during high glycolysis while promoting the proliferation, differentiation, and vascular sprouting of ECs; however, this process is pathological [28]. Thus, upon PFKFB3 knockdown, microglia lose the ability to promote pathological angiogenesis in OIR mice.

A recent study revealed the significant impact of nitric oxide (NO) on the metabolic remodeling of inflammatory macrophages through the regulation of PFKFB3 activity [92]. Notably, the absence of NO corresponds to diminished activity of PFKFB3. Conversely, NO-rich macrophages typically exhibit upregulated expression of PFKFB3. Consequently, a lack of NO leads to a reduction in PFKFB3 activity, influencing the metabolic remodeling of inflammatory macrophages. Within astrocytes, NO swiftly induces the upregulation of glycolysis, augmenting the synthesis of glycolytic byproducts within the cellular milieu [93]. The precise mechanism involves NO-mediated activation of AMPK phosphorylation, thereby enhancing the activity of PFK2.3 (PFKFB3), as described in the text. This activation, in turn, amplifies the yield of products in the glycolytic pathway. Notably, this regulatory mechanism assumes a pivotal role in governing astrocytic functions. Glycolytic flux in astrocytes is also regulated by the APC/C-Cdh1 pathway, thereby maintaining relatively stable PFKFB3 expression [93, 94]. In the face of Aβ amyloid stimulation, if PFKFB3 is inhibited, homeostasis function decreases, and neuronal cells then die [95]. The association between Müller cells and PFKFB3 has not been determined and represents a potential avenue for future research.

Inflammatory factors are predominantly secreted by glial cells in the retina; however, PFKFB3 also plays a crucial role in regulating inflammatory marker expression in ECs. Recent studies conducted this year indicate that when ECs are stimulated by inflammation, such as by exposure to bacterial lipopolysaccharide (LPS) [96], TNF-α [97], or IL-1β [98], the activity of PFKFB3, the main regulator of glycolysis, is significantly increased [99]. This increase in glycolytic flux is consistent with the increase in inflammatory responses in ECs. Correspondingly, when PFKFB3 is inhibited, inflammatory factor secretion by ECs is correspondingly reduced. From a mechanistic standpoint, the activation of PFKFB3 in ECs may implicate signaling pathways, such as the NF-κB pathway, in inflammation [97].

Hence, these findings underscore the pivotal role of PFKFB3-driven glycolysis in mediating inflammation in ECs and neovascular eye diseases. Consequently, this approach is anticipated to be efficacious for addressing neovascular ophthalmopathy. Targeting PFKFB3-driven glycolysis in ECs has emerged as a viable strategy for treating neovascular eye diseases.

### Effect of PFKFB3 on the regulation of the cell cycle and aging

PFKFB3 regulates the cell cycle in the nucleus. However, there are many regulatory kinases involved in cell cycle regulation, such as cyclin-dependent kinase (CDK) 4 and CDK 1, which are directly or indirectly linked to PFKFB3; CDK1 mediates the whole process of cell cycle initiation and termination, and CDK4 regulates cell cycle progression in the G1/S phase. CDK1 inhibition induces cell cycle arrest, and PFKFB3 functions as a regulator of CDK1 activity. Silencing PFKFB3 suppresses CDK1 activity, consequently stabilizing p27 protein levels and promoting cell cycle arrest at the G1/S phase [100]. Whereas PFKFB3 inhibits ubiquitin proteasome degradation mediated by the 90-Cdc37-CDK4 complex by binding to CDK4 at lysine 147 [101], CDK4 accelerates degradation upon mutation at this site. Simultaneous inhibition of PFKFB3 also promotes stabilization and accumulation of the p21 protein [102]. The APC/C-CdK1 pathway is involved in the mitotic transition and cell division, and this pathway can promote PFKFB3 ubiquitination and degradation to inhibit cell proliferation and control the number of cells in S phase [103]. The cell cycle is inextricably linked to cellular senescence, and high glucose-induced senescence in ECs is ameliorated by overexpression of PFKFB3 under pathologically high glucose conditions [81], but not further explored in cell cycle changes.

### Effect of PFKFB3 on the regulation of apoptosis

Knockdown of either PFKFB3 or MCT1 led to an increase in the percentage of apoptotic ECs, as demonstrated in the study by Hu et al. [104]. However, in another study, K472 acetylation of PFKFB3 after DNA damage was found to promote PFKFB3 accumulation in the cytoplasm and enhance the protective effect on cells [105], while knockdown of PFKFB3 further improved the apoptotic rate of cisplatin-induced injury. Inhibition of PFKFB3 not only promotes apoptosis but also concurrently inhibits the PI3K-Akt signaling pathway. This dual effect underscores the intricate relationship between PFKFB3 activity and cellular survival mechanisms [106]. Additionally, because of the AMPK-dependent phosphorylation of PFKFB3, oxidative respiration is replaced by glycolysis, promoting the survival and proliferation of ECs.

### Molecular regulatory mechanisms of PFKFB3

Numerous molecules play direct or indirect roles in regulating PFKFB3 expression. Specifically, inflammatory factors, including TNF-α and IL-1β as mentioned previously, directly stimulate the expression of PFKFB3 [97, 98], whereas knockdown of PFKFB3 can inhibit the expression of inflammatory factors. When ECs and other cells are subjected to hypoxia, oxidative stress, hyperosmolality and other stimuli, the expression of PFKFB3 is promoted through phosphorylation pathways, including the PR/ER, adenosine monophosphate-activated protein kinase (AMPK), PKA/PKC and other pathways [107–110]. Among them, HIF-1α, a hypoxic factor, is activated and promotes increased downstream VEGF expression [111]; subsequently, both HIF-1α and VEGF can target PFKFB3 and promote the expression of PFKFB3 [2], while HIF-1α is a powerful agonist of PFKFB3 [112]. Moreover, several microRNAs (miRs), such as miR-125a, miR-26a, and miR-26b, are involved in mediating the regulation of PFKFB3 and inhibiting its transcriptional activity by interacting with its 3’-UTR [113, 114].

In addition, certain long noncoding RNAs (lncRNAs) play crucial roles in the regulation of PFKFB3. Specifically, this article highlights the involvement of the lncRNA BCAR4, which functions as a downstream transcriptional target gene of YAP. The lncRNA BCAR4 directly governs the transcription of PFKFB3 by modulating histone acetylation, which is marked with H3K27ac [115]. This regulatory mechanism leads to the upregulation of PFKFB3 expression, consequently enhancing glycolytic processes and contributing to the progression of cancer. The long noncoding RNA (lncRNA) LINC00538, also known as YIYA, exerts its influence by augmenting CDK6-dependent phosphorylation of PFKFB3 through its interaction with the cell cycle protein CDK6 [116]. This molecular mechanism actively contributes to the promotion of cancer progression. Consequently, exploring the use of antisense oligonucleotides targeting lncRNAs has emerged as a promising avenue for developing novel therapeutic strategies.

In addition to VEGF, angiogenic factors such as FGF can also upregulate the expression of PFKFB3 and promote the generation and selection of vascular tip cells [2]. Notch signaling, which promotes the stalk cell phenotype, can inhibit the expression of PFKFB3 and reduce glycolysis-related factors [65]. While some factors associated with metabolism, such as FOXO1, which is highly enriched in ECs [117], are antagonists of retinal angiogenesis, they are able to substantially reduce glycolytic flux and lactate production. Whereas FOXO1 was shown to directly regulate PFKFB3, overexpressing PFKFB3 by knocking down FOXO1 reduced oxidative stress damage in ECs under high glucose conditions and promoted DNA damage repair [81].

FOXO1 also inhibits glycolysis by inhibiting C-MYC, thereby reducing the proliferation of ECs and retinal angiogenesis [117]. FOXO1 is a downstream molecule of Sirt1, and overexpression of Sirt1 inhibits EC dysfunction caused by high glucose concentrations through the silent information regulator 1 (SIRT1)-FOXO1-c-Myc signaling pathway [118]. Moreover, laminar shear stress (LSS) also affects ECs after angiogenesis, resulting in decreased glycolysis and mitochondrial activity, and silencing PFKFB3 by Krüppel-like factor 2 (KLF2) overexpression inhibits glycolysis [119, 120]. APC/C-Cdh1 was able to regulate the stabilization of PFKFB3 expression and prevent homeostatic imbalances resulting from PFKFB3 changes [94].

**Fig. 3:**
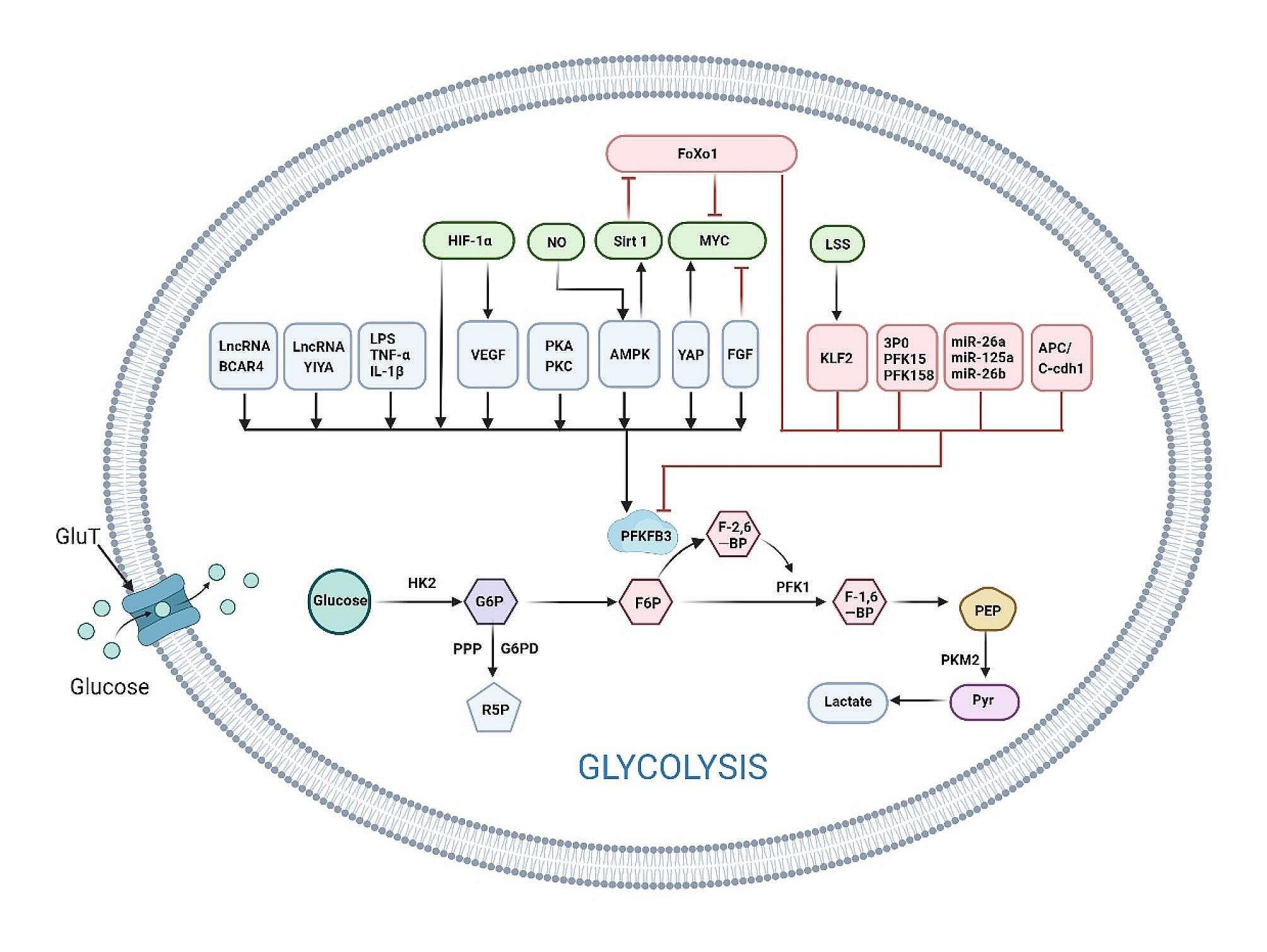
Glycolysis and the regulatory mechanism of PFKFB3 As mentioned earlier, glucose is converted into pyruvate through a series of crucial enzymatic reactions, and under hypoxic conditions, it is converted into lactate through enzymatic reactions. PFKFB3, an enzyme that plays a key role in glycolysis, is significantly affected by a wide range of factors, including inflammatory factors, miRNAs, long noncoding RNAs, growth factors and transcription factors. By modulating PFKFB3 activity through different mechanisms and pathways, these factors ultimately control glycolytic flux. Blue-boxed factors directly promote the expression of PFKFB3, while red-boxed factors directly inhibit its expression. The green-boxed factors are those directly or indirectly involved in the regulation of PFKFB3. This figure was created using Biorender (https://www.biorender.com/). GLUT: glucose transporter; HK2: hexokinase 2; G6PD: glucose6‑phosphate dehydrogenase; PFKFB3: phosphofructokinase‑2/fructose‑2,6‑bisphosphatase‑3; PFK1: phosphofructokinase‑1; PKM2: pyruvate kinase‑2; FGF: fibroblast growth factor; YAP: Yes‑associated protein; FOXO1: Forkhead Box O1; 3PO, 3‑(3‑pyridinyl)‑1‑(4‑pyridinyl)-2‑propen‑1‑one; KLF2, Krüppel‑like factor 2; LSS, laminar shear stress; LPS: lipopolysaccharide; IL-8: interleukin-8; IL-1β: interleukin-β; PKC: protein kinase C; HIF-1α: hypoxia-inducible factor; PKA: protein kinase A; AMPK: adenosine monophosphate-activated protein kinase; miR: microRNA; SIRT1: silencing information regulator 1; lncRNA: long noncoding RNA; NO: nitric oxide

The regulatory mechanism of PFKFB3, as illustrated in Fig. 3, is central to glycolysis. This review delves into the intricacies of cellular processes, encompassing inflammatory factor secretion, proliferation, migration, and DNA damage repair. These processes primarily intersect with the mechanisms and outcomes associated with angiogenesis. Understanding these interactions will lead to the identification of PFKFB3 as a crucial therapeutic target for conditions such as neovascular ophthalmopathy and other angiogenesis-related diseases.

### Drugs targeting PFKFB3

Ocular neovascular diseases are frequently treated with anti-VEGF therapy. However, this strategy is encumbered by various limitations, including side effects, limited efficacy, drug resistance, and other intricate challenges that pose difficulties in resolution. In recent years, alternative therapeutic avenues have surfaced, centering around medications designed to modulate the metabolism of ECs as a novel approach to the management of pathological neovascularization. Notably, directing interventions toward PFKFB3 has emerged as a promising alternative to conventional anti-VEGF therapy. We list the main types of PFKFB3 inhibitors sequentially in Table  1.

**Table 1:** Drugs targeting PFKFB3

Drug name	Clinical Status	Manufacturer	Current study
3PO	Preclinical	Advanced Cancer Therapeutics University of Louisville	[66]
PFK-15	Preclinical	Advanced Cancer Therapeutics University of Louisville	[106]
Phenoxyindole	Preclinical	AstraZeneca	[124]
AZ-67	Preclinical	Zhejiang University (Originator)	[125]
PFK-158	Phase I - NCT02044861	Advanced Cancer Therapeutics University of Louisville	[128]

3PO was the first PFKFB3 inhibitor to be widely used, and this inhibitory effect was achieved by competing with fructose 6-phosphate (F6P). 3PO was shown to inhibit neovascularization and promote normalization of angiogenesis in ocular diseases [66], with a similar effect on tumors [79], and improve the effect of chemotherapy on cancer ECs. In addition, 3PO can also inhibit the activation of ECs through a variety of inflammatory factors [121], and these effects can be independent of the effects produced by inhibiting glycolysis. Because 3PO cannot bind to PFKFB3 [122], its specificity is poor, and its high dose requirement leads to the delay in entering the clinical trial stage.

PFK15, an analog of 3PO, exhibited superior selectivity compared to 3PO. This molecule demonstrated a robust affinity for PFKFB3, resulting in enhanced target inhibition. Additionally, PFK15 has both anti-vascular normalizing and pro-vascular normalizing effects across diverse tumor types [106, 123]. In addition, phenoxyindole and AZ-67 have been widely studied in preclinical studies. Through a series of chemical syntheses and screens, phenoxyindole derivatives were shown to have high selectivity for PFKFB3 and to directly interact with ATP [124], but its effect in vivo has not been verified. AZ-67 was shown to significantly inhibit angiogenesis in vivo at low doses and specifically bind to PFKFB3 [125], while AZ-67 also promoted normalization of the PFKFB3 content in neurons and also had a potential therapeutic effect on neurodegenerative diseases [126].

PFK158, a derivative of PFK15, represents a pioneering PFKFB3 inhibitor that has progressed to phase 1 clinical trials in humans (clinical trial: NCT02044861). In contrast to 3PO and PFK15, this compound has heightened specificity and efficacy against PFKFB3 and improved tolerability in animal models. Numerous studies have additionally validated the robust antitumor activity of PFK158, particularly in the context of antiangiogenic agents [127, 128]. PF158 was produced by optimizing 3PO as well as PFK15 and is now the only drug to enter phase I clinical trials, demonstrating that it is a safe and effective antiangiogenic treatment. Many preclinical studies on PFKFB3 inhibitors have been conducted, and these agents are promising in combination with anti-VEGF agents or alone for the treatment of neovascular eye disease in the future.

## Discussion

PFKFB3 serves as a crucial regulatory enzyme in glycolysis and oversees the proliferation of ECs and the development of vascular sprouting. It accomplishes this by controlling the formation of filopodia/lamellipodia, the direction of migration, and the intricate connection between EC functions and metabolism. Notably, both ECs and glial cells within the retina rely on glycolysis and are thus susceptible to metabolic disorders. This highlights the occurrence of metabolic crosstalk between these cells. Furthermore, glycolysis constitutes a relatively small portion of the human body’s metabolic activities, suggesting that targeted glycolysis therapy is a safe and viable approach. Within the scope of this paper, we also investigated the therapeutic effects of targeting PFKFB3 in the treatment of neovascular eye diseases such as DR, AMD, and ROP. However, the protective mechanisms induced by this targeting strategy vary across these diseases.

In addition to the pivotal role of PFKFB3, a crucial enzyme, numerous enzymes and proteins participate in glucose metabolism, warranting further investigation. Notably, enzymes such as HK2, PKM2, GLUT1, and other members of the PFKFB family have the potential to significantly influence vascular homeostasis regulation. Furthermore, PFKFB3 not only affects angiogenesis and related mechanisms through glycolysis control but also plays a vital role in the interaction of multiple molecular mechanisms. Similarly, inhibitors of PFKFB3 possess protective properties that extend beyond PFKFB3 expression regulation, encompassing other mechanisms and pathways.

In numerous studies exploring the association of PFKFB3, several unresolved issues persist. Pharmaceuticals targeting PFKFB3 are still in the early stages of development, and no relevant drugs have entered clinical Phase II studies. Consequently, there is a pressing need to investigate the safety, dosage, and mechanism of action of these agents. While numerous studies have explored various mechanisms in ECs using different disease models, these mechanisms remain unexplored in animal models of ocular neovascularization. Specifically, the effects of targeting PFKFB3 on inflammatory secretion, apoptosis, and pyroptosis in ECs have not been examined in this context. Notably, targeting PFKFB3 may yield different effects depending on the pathological or physiological conditions. For example, while overexpression of PFKFB3 increases oxidative stress-induced damage in normal ECs, it protects ECs under high glucose (HG) conditions. This observation has significant implications for future studies. The impact of metabolic crosstalk and signaling mechanisms between ECs and surrounding glial cells, among other factors, remains an area of investigation requiring more in-depth exploration. Currently, these aspects have not been fully elucidated. Finally, the inhibition of PFKFB3 has been shown to normalize angiogenesis in tumors. However, its effect on ocular angiogenesis is limited by its ability to reduce angiogenesis in ocular diseases. Moreover, this approach does not truly demonstrate the normalization of ocular angiogenesis but rather defines a decrease in angiogenesis as promoting the normalization of blood vessels.

## Conclusion

Many key enzymes involved in glycolysis have been discovered, but only a few have been thoroughly studied and investigated. In particular, PFKFB3 has garnered significant attention in recent years as a key enzyme that researchers worldwide have been striving to comprehend. Targeting PFKFB3 has yielded promising results in the treatment of vascular-related diseases, such as cancer, pulmonary hypertension, and atherosclerosis. Additionally, this enzyme represents a novel target for neovascular eye disease treatment. Although neovascular eye disease is considered the most prevalent and detrimental ocular fundus disease, it currently lacks a cure, necessitating the exploration of new therapeutic approaches. With the development of various drugs targeting PFKFB3 and their gradual entry into clinical trials, targeting PFKFB3-mediated glycolysis has emerged as a promising therapeutic method for neovascular eye disease in the future.

## Data Availability

Not applicable.

## Abbreviations

DR: Diabetic retinopathy
PDR: Proliferative diabetic retinopathy
NPDR: Non-proliferative diabetic retinopathy
AMD: Age-related macular degeneration
ROP: Retinopathy of prematurity
VEGF: Anti-vascular endothelial growth factor
PFKFB3: Phosphofructo-2-kinase/fructose-2,6-bisphosphatase3
ATP: Adenosine triphosphate
ECs: Endothelial cells
PFK-1: Phosphofructokinase-1
F2,6BP: Fructose 2,6-bisphosphate
RNV: Retinal neovascularization
TCA: Tricarboxylic acid
OXPHOS: Oxidative phosphorylation
RPE: Retinal pigment epithelium
MCT1: Monocarboxylate transporter 1
HK2: Hexokinase 2
PKM2: Pyruvate kinase muscle isozyme M2
GLUT1: Glucose transporter 1
PDGF: Platelet-derived growth factor
PRAGMs: Pathological retinal angiogenesis glycolytic microglias
PDGFB: Platelet-derived growth factor subunit B
TGF-1: Transforming growth factor-β1
VEGFR-1: Vascular endothelial growth factor receptor-1
Dll4: Delta-like 4
NICD: Notch intracellular domain
AGEs: Advanced glycation end products
PKC: Protein kinase C
HIF-1α: Hypoxia-inducible factor
Ang: Angiopoietin
FGF: Fibroblast growth factor
VEGFR2: Vascular endothelial growth factor receptor-2
ROS: Reactive oxygen species
DNA: Deoxyribonucleic acid
PARP: Poly ADP-ribose polymerase
NAD: Nicotinamide adenine dinucleotide
GAPDH: Glyceraldehyde-3-phosphate dehydrogenase
MMPs: Matrix metalloproteinases
NADPH: nicotinamide adenine dinucleotide phosphate
NOX: Nicotinamide adenine dinucleotide phosphate oxidase
NF-κB: nuclear factor κB
Cyct-c: Cytochrome c
MCP-1: Monocyte chemoattractant protein-1
MIP-1: Macrophage inflammatory protein-1
TNF-α: Tumor necrosis factor-α
IL-6: Interleukin-6
IL-8: Interleukin-8
IL-1β: Interleukin-β
OIR: Oxygen-induced retinopathy
CNV: Choroidal neovascularization
Adora2a: Adenosine A2a receptor
YAP: Yes-associated protein
EGF: Epidermal growth factor
HUVEC: Human umbilical vein endothelial cell
HRMEC: Human retinal microvascular endothelial cell
FOXO1: Forkhead box protein O1
MRN: Mre11-Rad50-Nbs1
ATM: Ataxia telangiectasia mutated
RGCs: Retinal ganglion cells
endoMT: Endothelial mesenchymal transformation
ECM: Extracellular matrix
Aβ: Amyloidβ
LPS: Lipopolysaccharide
CDK: Cyclin-dependent kinase
AMPK: Adenosine mono-phosphate-activated protein kinase
miR: microRNAs
SIRT1: Silence information regulator 1
LSS: Laminar shear stress
KLF2: Krüppel-like factor 2
F6P: Fructose 6-phosphate
HG: High glucose
lncRNA: Long noncoding RNA
NO: Nitric oxide
